# The influence of parent–child relationships on the learning adaptability of left-behind children: the mediating role of peer attachment and the moderating role of separation duration

**DOI:** 10.3389/fpsyg.2023.1108993

**Published:** 2023-07-27

**Authors:** Ning Chen, Keyun Zhao, I-Hua Chen, Guanling Liu

**Affiliations:** ^1^School of Communication, Qufu Normal University, Rizhao, Shandong, China; ^2^Chinese Academy of Education Big Data, Qufu Normal University, Qufu, Shandong, China

**Keywords:** left-behind children, parent–child relationships, peer attachment, learning adaptability, separation duration

## Abstract

Studies have revealed the influence of parent–child relationships on the learning adaptability of left-behind children. However, the researchers have not explored the mechanisms underlying the parent–child relationships of left-behind children. The purpose of this study was not only to examine the mediating role of peer attachment in the relationship between parent–child relationships and learning adaptability but also to explore the moderating variable of separation duration in the relationship between parent–child relationships and peer attachment. The study examined 1,555 left-behind children and found that, after controlling for gender and grade, parent–child relationships positively predicted learning adaptability; peer attachment mediated the relationship between parent–child relationships and learning adaptability, and separation duration moderated the effect of parent–child relationships on peer attachment. The study reveals the importance of parent–child relationships and peer attachment in the growth and development of left-behind children, which is important for the improvement of left-behind children’s learning adaptability.

## 1. Introduction

With rapid economic development, a large number of rural laborers have chosen to move from rural to urban areas in search of more employment opportunities, resulting in a large number of children staying at home on their own. This behavior has a negative impact on children’s psychological, emotional and behavioral development and can lead to serious problems such as academic performance (Li et al., 2015; Wu and Zhang, 2017), physical health (Ban et al., 2017; Lei et al., 2018), and behavioral disorders (Wen et al., 2019). As a part of social development, paying attention to the growth of left-behind children is not only related to their healthy development but also helps to improve the social support system in children’s development and lay a solid foundation for social development. Left-behind children are children who are left at home when one or both parents go out to work and who need the care of other relatives or entrusted persons at the compulsory education stage (Wang et al., 2013). The left-behind children in rural areas in this study are children aged 6–16 years old who have one or both parents working in the city and who are left to live on their own in rural areas, usually living with one parent or other relatives.

According to Mc Master’s family function model theory (Miller et al., 2000), the basic function of the family is to provide certain environmental conditions for the healthy development of family members, and the level of family closeness affects the function of the family, and family communication as a facilitating factor can influence the level of family closeness. The parent–child relationships is an important expression of the closeness of the family and its strengths and weaknesses can have a direct impact on children’s performance in learning. The strong emotional bond between parent and child is an important foundation for their emotional and affective development and has a significant impact on the development of other personal emotions in children.

In addition to the family environment, school is an important place for children to grow and develop, and teachers’ enthusiasm in teaching can have a significant impact on children’s achievement development (Moe et al., 2021). Furthermore, research has shown that peer support in the school environment can alleviate the isolation of left-behind children and enhance their sense of belonging, thus contributing to their psychological development (Cohen et al., 2009). Peer support and encouragement in the learning process can help children to form good study habits in the learning process, thus contributing to their academic achievement (Lee and Simpkins, 2021; Pap et al., 2021). Moreover, the care and affection of peers in daily life can give children confidence and warmth, which plays a key role in compensating for the lack of family support functions for left-behind children (Tapia-Fonllem et al., 2020; Leurent et al., 2021). In addition, learning adaptability, as an important characteristic of individual development, is an important guarantee for children to overcome difficulties in the learning process, adapt to the learning environment and make academic progress. Good learning adaptability contributes to children’s psychological well-being and interpersonal relationships, which are essential for their growth and development (Stockinger et al., 2021; Şahin and Gülşen, 2022).

Therefore, this study explores the factors and mechanisms that influence the learning adaptability of left-behind children and strengthens the main responsibility of family guardianship and the awareness of social care and support, which is of great significance to improve the academic achievement of left-behind children and promote their growth and development.

### 1.1. The relationship between parent–child relationships and learning adaptability

The parent–child relationships, as an important aspect of the family environment, plays a crucial role in the development of children (Hong et al., 2019). According to Bowlby’s attachment theory (Bowlby, 1982), the attachment relationship between parents and children can have an impact on the development of children’s personality and social understanding. Therefore, a good parent–child relationships in the family environment is conducive to the formation of good learning habits and can provide more support and assistance to the child, resulting in more engagement in learning (Chen et al., 2018).

Learning adaptability refers to an individual’s ability to make appropriate cognitive, behavioral, and emotional adjustments in the face of uncertainty and novelty (Burns et al., 2018). Adaptability is somewhat linked with the capability to adopt effective strategies (De Beni and Moè, 2003). Good learning adaptability can increase students’ engagement in their studies and improve their sense of achievement and well-being (Burns et al., 2018). Previous research has shown that parent–child relationships, particularly parental support, predict children’s behavior and attitudes (Xu et al., 2021). In other words, parental teaching and care in learning and life can influence the production of children’s behavior and predispose children to develop positive attitudes towards life and learning, which is crucial to child development (Qi and Wu, 2020). At the same time, parent–child relationships play a crucial role in supporting students’ learning process. Harmonious parent–child relationships can enhance children’s confidence in facing various problems and challenges in learning, thus improving their development of learning abilities (Mulyadi et al., 2016).

Left-behind children are provided with windfalls of abundant resources as a result of the higher city wages remitted by their migrant parents, while facing the disruption of family stability by becoming parentless (Chang and Lu, 2018). The disruption of family stability makes parent–child communication, parent–child education, and parent–child interaction less frequent, which will have a significant negative impact on children’s non-cognitive abilities (Zhao and Chen, 2022). It has been shown that the development of children’s non-cognitive skills has a positive effect on the improvement of students’ academic performance (Durlak et al., 2011). Therefore, this study proposes hypothesis H1: Parent–child relationships positively predict adolescent learning adaptability.

### 1.2. The mediating role of peer attachment

Peer attachment, as the first step in adolescent interpersonal development, refers to the mutual feelings of closeness and warmth, and support that adolescents develop with their peers (Ji et al., 2022). The attachment relationship between adolescents and their parents and peers is a core element in their growth and development. Modern attachment theory suggests that children can use ambivalent attachment (to increase demand signals) to maximize available investment in situations where parents are willing but unable to consistently invest in their offspring (Del Giudice and Belsky, 2010). Ambivalent attachment predicts pro-social resource control (Chen and Chang, 2012), individuals can access to resources in the social group through cooperation or reciprocity. Left-behind children tend to develop and adopt an ambivalent attachment style due to their lack of security. The attachment goals of left-behind children are more inclined toward their peers as their parents work outside the home (Lim, 2020). The higher the intimacy and acceptance between parents and children, the better the adolescent’s peer attachment relationships (Cook et al., 2012). Specifically, a harmonious and cordial parent–child relationships allows for smoother parent–child communication and enables parents to act as supportive role models who promote positive relationships with peers when they provide appropriate supervision for their adolescents (Lim, 2020).

In addition, peer support is crucial to students’ school engagement (Wang and Eccles, 2013). Peer attachment, as an extension of the quality of the parent–child relationships, has the ability to reduce the anxiety and fear associated with parent–child separation and has a positive impact on the social as well as personality development of left-behind children (Boliang et al., 2005; Goossens, 2020). In particular, left-behind children whose parents work outside of the home are in great need of peer support. A harmonious relationship between parents and children provides children with a greater sense of security and trust, which can have a significant impact on children’s peer interaction. For left-behind children, the fact that their parents work outside the home is not conducive to the establishment of parent–child relationships. However, the positive interactions with peers can lead to greater behavioral and emotional engagement in school and can create a positive classroom atmosphere, thereby increasing participation in the classroom. At the same time, peers play a key role in shaping students’ behavior by providing emotional support, companionship, and motivation, and can contribute to students’ academic achievement by improving their attitudes and abilities to learn (Ansong et al., 2017).

According to ecosystem theory (Bronfenbrenner, 1979), the family system and the peer system are important influences on individual development and adaptation, and the two systems interact to promote the growth and development of children. Therefore, parent–child relationships, as the foundation of the family environment for good social adjustment in adolescents, can facilitate the establishment of peer attachment relationships. As a key factor influencing adolescents’ academic achievement, peer relationships can play a positive predictive role in adolescents’ learning adaptability (Ansong et al., 2017). Based on this, this study proposes hypothesis H2: Peer attachment mediates the relationship between parent–child relationships and the learning adaptability of left-behind children.

### 1.3. The moderating effect of separation duration

As a core feature of the special group of left-behind children (Ling et al., 2015), the amount of separation duration that comes with parent–child separation is an important indicator of the family structure of left-behind children, which has a very important impact on the physical and mental health development of left-behind children. The length of separation duration refers to the time when one or both parents are away from their children in order to reduce the economic pressure on the family (Ling et al., 2012). It has been shown that the length of separation duration is a valid predictor of children’s emotions and problem behaviors and that the longer parents are separated from their children, the greater the negative impact on children. Furthermore, the length of parent–child separation can influence the emotional connection between parent and child to some extent (Zhang et al., 2022).

Specifically, the reduced frequency of parent–child communication caused by parents going out to work will weaken the parent–child relationships of left-behind children, gradually dilute the attachment role of left-behind children to their parents, and promote the children’s attachment object from parents to peers (Fan and Lu, 2020). As the premise and basis for individuals to get along harmoniously with others, interpersonal skills play an important role in the process of left-behind children’s attachment object conversion. Compared with children left behind for a long time, children left behind for a short time are less affected by the adverse situation of lack of family emotion. Parents can still give children higher encouragement and support, so as to enhance the confidence of individuals in social communication, and the development process of their interpersonal skills is smoother. On the contrary, children left behind for a long time due to long-term separation from their parents will lead to long-term accumulation of pressure (Claes et al., 2015), easy to produce low self-esteem, withdrawn psychology and negative emotions, not conducive to the development of interpersonal skills, and then affect the parent–child relationship on peer attachment. Therefore, separation duration can play a moderating role between parent–child relationships and peer attachment. As the length of stay increases, the time available for normal communication and interaction between parents and children is affected. The longer the period of stay, the more the children lack deep psycho-emotional contact with their parents, which can lead to a decrease in their social interaction skills and thus affect their peer relationship building.

Based on this, the study proposes hypothesis H3: The separation duration plays a moderating role between parent–child relationships and peer attachment. That is to say, the role of parent–child relationships in influencing peer attachment of left-behind children varies according to the length of time children are left-behind.

### 1.4. Summary

**Figure 1:**
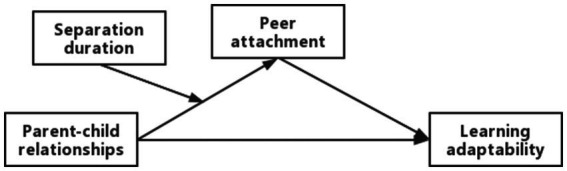
Conceptual model diagram.

This study sought to construct a moderated mediation model to understand the role of parent–child relationships in influencing learning adaptability. As shown in Figure 1, the objectives of the study were: (a) to determine whether the parent–child relationships was a positive predictor of left-behind children’s learning adaptability; (b) determine whether peer attachment mediates the relationship between parent–child relationships and learning adaptation; (c) determine whether separation duration regulates the indirect effects of parent–child relationships on learning adaptation.

## 2. Research methodology

### 2.1. Subjects

The survey was conducted using a whole-group sampling method, and a county in Gansu Province with a high number of left-behind children was selected. The studies involving human participants were reviewed and approved by Qufu Normal University of ethics committee. Written informed consent to participate in this study was provided by the participants’ participants legal guardian/next of kin. The study fully complied with all the requirements of the ethical and consent procedures, and the relevant research content would not cause harm to the left-behind children. Moreover, the study obtained informed consent from the legal guardians/close relatives of the left-behind children prior to data collection. In addition, to protect the privacy of the left-behind children, the study was conducted by surveying a sample of all children and selecting a sample of left-behind children for the study, and the process of the study fully respected the wishes and interests of the left-behind children. Based on this, the study selected several primary and secondary schools in the county, with the consent of the local education bureau, and sampled 4,494 children by whole group of classes.

4,437 valid questionnaires were obtained after excluding questionnaires with incomplete information, with a valid questionnaire return rate of 98.7%. In the basic condition survey, the questions “At present, does the father or mother work outside the home?” and “How long has the father or mother worked outside the home continuously?” were designed to eliminate the sample of children who were not left-behind children, and finally, 1,555 samples of left-behind children were obtained. Table 1 shows in the valid samples, 808 (52%) children were left-behind girls and 747 (48%) children were left-behind boys; in terms of grade distribution, 666 (42.8%) children were left-behind in grades 4 and 5, 513 (33.0%) children in grades 6 and 7, and 376 (24.2%) children in grades 8 and 9; 599 (38.5%) children were left-behind for less than 6 months, and 679 (43.7%) children were left-behind for 6 to 12 months. 679 (43.7%) children stayed for less than 6 months and 277 (17.8%) children stayed for 12 months or more.

**Table 1:** Demographic statistic.

Variables	Category	Number	Percentage	Variables	Category	Number	Percentage
Gender	Male	747	48.0%	Are you an only child?	Yes	147	9.5%
	Female	808	52.0%		No	1,408	90.5%
Grade Level	Grade 4 and 5	666	42.8%	separation duration	Up to 6 months	599	38.5%
	Grade 6 and 7	513	33.0%		6–12 months	679	43.7%
	Grade 8 and 9	376	24.2%		More than 12 months	277	17.8%

### 2.2. Research tools

#### 2.2.1. Parent–child relationships scale

The Parent–Child Relationships Scale is based primarily on the Parent–Child Intimacy Scale developed by Buchanan et al. (1991). The adapted scale contains two subscales with a total of 10 items: Father-Child Relationship (5 items, e.g., I get on really well with my dad) and Mother–Child Relationship (5 items, e.g., I get on really well with my mum). The questions on the Father-Child Relationship Scale are the same as those on the Mother–Child Relationship Scale, except that they are referred to differently. Each item on the scale is scored on a 5-point Likert scale (1 = “not at all,” 5 = “fully”). The mean value of the overall scale score indicates the subject’s perceived parent–child relationships status, with higher scores indicating a more positive parent–child relationships. It has been demonstrated that the scale has good reliability and validity and is a valid measure of the subjects’ parent–child relationships status (Tian et al., 2018). The Cronbach’s alpha for the whole scale of Parent–child Relationships Scale was 0.840.

#### 2.2.2. Learning adaptability scale

The Learning Adaptability Scale was adapted from the Learning Adaptability Scale developed by Feng et al. (2006). The adapted scale includes two dimensions of learning attitude (e.g., “I never ignore difficulties in learning.”) and learning ability (e.g., “I can use what I have learned to solve new problems.”), with a total of nine items. Each item is scored on a 5-point Likert scale (1 = “not at all,” 5 = “fully”). The total mean score of the items on the scale represents the subject’s level of learning adaptability, with higher scores representing greater learning adaptability. It has been shown that the scale has good reliability and validity, and can effectively measure the learning adaptability of the respondents (Zeng et al., 2008). The Cronbach’s alpha for the whole scale of Learning Adaptability Scale was 0.908.

#### 2.2.3. Peer attachment scale

Referring to the peer attachment questionnaire in the Inventory of Parent and Peer Attachment (IPPA) developed by Armsden and Greenberg (1987), this study developed a peer attachment scale for left-behind children through both peer communication and peer support. The scale consists of six items, for example, “My friends often help me when I am in trouble.” Each item on the scale is scored on a 5-point Likert scale (1 = “not at all,” 5 = “fully”). The mean of the summed items on the scale was the peer attachment score, with higher scores indicating stronger relationships with friends in academic life. In this study, Cronbach’s alpha coefficient for the self-administered scale was 0.798, with good reliability.

#### 2.2.4. Separation duration variables

Firstly, the study identified three types of children’s parents working outside the home based on the question “At present, does either the father or the mother work outside the home”: (1) the father works outside the home; (2) the mother works outside the home; (3) both parents work outside the home. Secondly, the study determined the length of separation duration children were left behind based on their parents’ absence. Specifically, when the child’s father worked outside the home, the duration of the child’s separation was determined according to the question “How long did the father stay outside the home continuously?” When the child’s mother is working, the child’s length of separation duration is determined by the question “How long has the mother been away continuously?” When both parents work, the child’s length of separation is determined by the question “How long is the father away continuously?” and “How long is the mother away continuously?” In this case, separation duration is equal to the longer of the father’s and mother’s consecutive time away from home. In addition, the study reassigned a combination of the duration of stay corresponding to the three types of parental work outside the home to a new variable separation duration.

#### 2.2.5. Control variables

The study used the individual characteristics of the respondents as control variables to exclude their influence on the findings. The control variables included the gender (boys = 1, girls = 2) and grade level (grades 4 and 5, grades 6 and 7, grades 8 and 9) of the left-behind children.

### 2.3. Statistical analysis

This study used the statistical analysis software SPSS 24.0 to analyze and process the data. Firstly, the study conducted a common method bias test as well as a multicollinearity diagnosis. Secondly, descriptive statistics between variables were conducted to understand the demographic characteristics of the respondents, and Pearson correlation analysis was used to test the relationship between the variables. Thirdly, the study used Model 4 of the SPSS macro program suggested by Hayes (2017) to test the mediating role of peer attachment in the relationship between parent–child relationships and learning adaptability. As well as controlling for two variables, gender and grade, the study tested the moderation of separation duration using Model 7 of the macro procedure. Finally, to gain insight into the moderation of the moderating variable separation duration on the model, the study used the Johnson-Neyman method (Dearing and Hamilton, 2006) to conduct a simple slope test, which provided insight into the moderating effect of different separation duration on the model.

## 3. Results

### 3.1. Common method bias and multicollinearity test

The study used respondents’ self-reporting to collect data, which may lead to common method bias issues. Therefore, the study controlled for bias by designing reverse questions and using anonymous surveys at the questionnaire design and distribution stage on the one hand; on the other hand, the study used the Haman one-way method to conduct a common method bias test at the data analysis stage (Podsakoff et al., 2003). The results of the study showed that the KMO and Bartlett’s sphericity test results were 0.926 (*p* < 0.000). In addition, the cumulative variance explained by the first factor with an eigenroot greater than 1 extracted based on the principal component analysis method was 38.92%, which was below the critical 40% threshold (Liu and Wei, 2020). This indicates that the systematic error caused by the common method bias was not serious and the data analysis results were reliable.

In addition, to avoid the effect of multiple co-linearity problems between variables on the study results, the study performed multiple co-linearity diagnoses in the regression analysis. Based on the results, it can be seen that the variance inflation factor (VIF) value is less than 2 and the tolerance is greater than 0.5, indicating that there is no multicollinearity problem among the study variables (Chennamaneni et al., 2016).

### 3.2. Descriptive statistics and correlation analysis

Table 2 shows the results of the descriptive statistics and correlation analysis between the variables studied. Based on the results, the four variables of the parent–child relationships, peer attachment, learning adaptability, and separation duration were correlated with each other and the correlation coefficient was less than 0.5. Parent–child relationships, peer attachment, and learning adaptability were positively correlated, while separation duration was negatively correlated with all three variables. This result indicates that the parent–child relationships and peer attachment of left-behind children decreases as the separation duration increases and that the learning adaptability of left-behind children also decreases as the separation duration increases. In addition, the moderating variable of gender was positively correlated with learning adaptability, and the moderating variable of grade level was negatively correlated with all four study variables. The results of the study provide preliminary evidence for the hypothesis.

**Table 2:** Descriptive statistics and correlation analysis between variables.

Variables	1	2	3	4	5	*M (SD)*
1. Gender						
2. Grade	0.020					
3. Parent–child relationships	0.006	−0.130^***^				3.911 (0.843)
4. Peer attachment	0.033	−0.066^**^	0.429^***^			3.897 (0.844)
5. Learning adaptability	0.058^*^	−0.069^**^	0.463^***^	0.439***		4.075 (0.683)
6. Separation duration	0.002	−0.050^*^	−0.091^***^	−0.075^**^	−0.072^**^	1.793 (0.722)

^*^p < 0.05; ^**^p < 0.01; ^***^p < 0.001.

### 3.3. Mediation model testing

To test the direct effect of the parent–child relationships on learning adaptability, the study first controlled for two variables, gender and grade level. According to Model 1 in Table 3, the parent–child relationships was a significant positive predictor of learning adaptability after controlling for gender and grade (*β* = 0.374, *SE* = 0.018, *p* < 0.001), indicating that left-behind children with good parent–child relationships are more adaptive in their learning. Therefore, hypothesis H1 was tested. To test the mediating role of peer attachment between parent–child relationships and learning adaptation, the study used Model 4 in the PROCESS macro program for the validation of the mediation model (Hayes, 2017). As shown in Model 2, parent–child relationships had a significant positive predictive effect on peer attachment after controlling for control variables (*β* = 0.427, *SE* = 0.023, *p* < 0.001), indicating that left-behind children with close parent–child relationships were more likely to develop an attachment to their peers when spending time with them. In Model 3, when peer attachment was included as a mediating variable in the model, it was able to positively predict learning adaptation (*β* = 0.237, *SE* = 0.019, *p* < 0.001). Moreover, the indirect effect of the parent–child relationships on learning adaptability through peer attachment was significant (*β* = 0.273, *SE* = 0.019, *p* < 0.001), indicating that the parent–child relationships not only predicted learning adaptability directly but also had an effect on learning adaptability through peer attachment relationships. Therefore, hypothesis H2 is verified.

**Table 3:** Examining the mediating role of peer attachment on learning adaptability.

Predictors	Model 1(Learning adaptability)			Model 2(Peer attachment)			Model 3(Learning adaptability)		
	*β*	*SE*	*t*	*β*	*SE*	*t*	*β*	*SE*	*t*
Constant	2.513^***^	0.097	26.021	2.168^***^	0.122	17.802	2.000^***^	0.101	19.765
Gender	0.076^*^	0.031	2.477	0.052	0.039	1.330	0.064^*^	0.029	2.177
Grade	−0.008	0.019	−0.429	−0.012	0.024	−0.470	−0.006	0.019	−0.303
Parent–child relationships	0.374^***^	0.018	20.382	0.427^***^	0.023	18.471	0.273^***^	0.019	14.102
Peer attachment							0.237^***^	0.019	12.307
*R^2^*	0.218			0.185			0.287		
*F*	143.970***			117.231***			156.317^***^		

*N* = 1,555; ^*^p < 0.05; ^**^p < 0.01; ^***^p < 0.001.

### 3.4. Moderated mediation model testing

To test whether the separation duration had an influential effect on the association between parent–child relationships and peer attachment, the study used Model 7 from the PROCESS macro program to test this (Hayes, 2017). According to Model 1 in Table 4, parent–child relationships were positively associated with peer attachment after controlling for the variables gender and grade (*β* = 0.429, *t* = 18.453, *p* < 0.001), and this association was moderated by the moderating variable separation duration. Also, to ensure the reliability of the results of the data analysis, the study set the variables to a mean-centered centrality operation (Castro-González et al., 2019). On this basis, the interaction term between the parent–child relationships and separation duration likewise had a significant effect on the variable of peer attachment (*β* = −0.090, *t* = −2.866, *p* < 0.01). The results suggest that the length of separation duration moderates the predictive effect of the parent–child relationships on peer attachment.

**Table 4:** Examining the moderating effect of separation duration on peer attachment.

Predictors	Model 1(Peer attachment)			Model 2(Learning adaptability)		
	*β*	*SE*	*t*	*β*	*SE*	*t*
Constant	3.855^***^	*0.093*	*41.677*	3.082^***^	*0.102*	*30.234*
Parent–child relationships	0.429^***^	0.023	18.401	0.272^***^	0.019	14.040
Peer attachment				0.237^***^	0.019	12.306
Separation duration	−0.044	0.027	−1.642			
Parent–child relationships×Separation duration	−0.091*	0.032	−2.874			
Gender	0.046	0.039	1.192	0.064^*^	0.029	2.182
Grade	−0.005	0.012	−0.470	−0.004	0.009	−0.484
*R^2^*	0.191			0.288		
*F*	72.896^***^			156.367^***^		

*N* = 1,555; ^*^p < 0.05; ^**^p < 0.01; ^***^p < 0.001.

**Figure 2:**
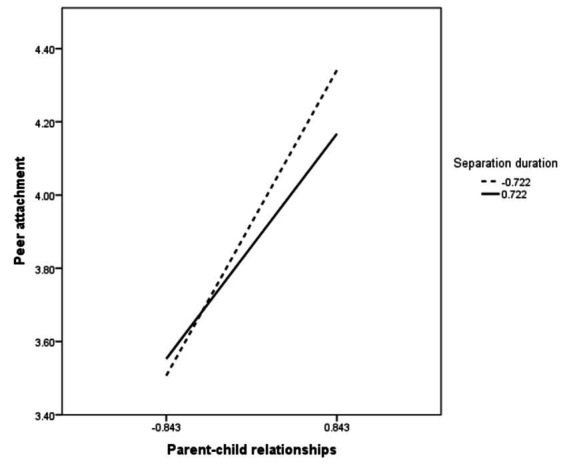
The moderating effect of separation duration in the parent–child relationships and peer attachment.

To further understand the moderating mechanisms of separation duration, and in particular the effect of parent–child relationships on peer attachment at different levels of separation duration, the study used a simple slope analysis using a point selection method. The mean of the scores for separation duration, plus one standard deviation of the data was defined as the high subgroup, and the mean of the scores for the survey sample minus one standard deviation of the data was defined as the low subgroup for statistical analysis. Figure 2 indicate that the positive predictive effect of parent–child relationships on peer attachment diminishes as the length of time children remain behind increases. Based on the above analysis, hypothesis H3 was tested.

In addition, the study further verified that the indirect effect of the parent–child relationships on learning adaptability through peer attachment was moderated by the separation duration through bias-corrected percentile. According to Table 5, the study calculates the mediating effect of peer attachment of left-behind children on parent–child relationships and learning adaptability when the separation duration score is three levels: the mean minus one standard deviation, the mean, and the mean plus one standard deviation. The results indicated that the effect of peer attachment as a mediating variable was diminishing as the separation duration changed from mean minus standard deviation [*Effect* = 0.117, 95% *CI* (0.089, 0.148)] to mean plus standard deviation [*Effect* = 0.086, 95% *CI* (0.063, 0.113)]. In other words, the length of separation duration that left-behind children are separated from their parents can affect children’s learning adaptability by influencing the relationship between parent–child relationship and peer attachment. Moreover, the longer the separation duration, the weaker the influence of parent–child relationship on learning adaptability through peer attachment.

**Table 5:** Mediating effects of peer attachment at different levels of separation duration.

Level of separation duration	*Effect*	*BootSE*	*BootLLCI*	*BootULCI*
Mean-1SD	0.117	0.015	0.089	0.148
Mean	0.102	0.013	0.079	0.128
Mean + 1SD	0.086	0.013	0.063	0.113

Mean is the average score of staying time; SD is the standard deviation of left-behind time score.

## 4. Discussion

The main objective of this study is to examine whether parent–child relationships can have an impact on the learning adaptability of left-behind children. Therefore, a moderated mediation model with the parent–child relationships as the independent variable, peer attachment as the mediating variable, separation duration as the moderating variable, and learning adaptability as the dependent variable was tested with left-behind children aged 6–16. The results showed that peer attachment mediated the relationship between the parent–child relationships and learning adaptability and that the indirect relationship between the parent–child relationships and learning adaptability was moderated by the separation duration.

### 4.1. The mediating role of peer attachment

The parent–child relationships is the first intimate relationship experienced by an individual as he or she grows up, and it has a significant impact on the development and adjustment of children and adolescents (Ma et al., 2021). This study found that the parent–child relationships of left-behind children positively predicted their learning adaptability, i.e., the more amicable the parent–child relationships was during the time the child was left-behind, the more conducive it was to the child’s improved learning ability and personal growth and development. This is similar to the findings that parent–child relationships, as an important variable in the family environment, can influence children’s academic achievement and cognitive development to some extent (Yeung et al., 2002). Harmonious parent–child relationships are an important component of children’s healthy physical, psychological and cognitive development, and are also a factor that cannot be ignored in promoting children’s good attitudes towards learning and their ability to learn, and their impact on children’s learning adaptability is crucial (Jeynes, 2007).

According to the process-person-context model of ecosystem theory (Bronfenbrenner, 1992), the environment in which people live is crucial to their growth and development, primarily because developing people and their environment can interact and influence each other. The child’s family members and members of other organizations, such as those at school and peer groups, act as constructors of the child’s developmental environment and interact with each other to influence the development of the young person (Yendork, 2020). Therefore, parent–child relationships and peer attachments, as intimate relationships essential to children’s growth and development, can have a positive and important impact on children’s healthy growth and academic achievement. The emotional absence of left-behind children due to their parents working outside the home often enhances their emotional connection with their peers (Wen and Lin, 2012). Peer relationships are closely related to individual engagement in learning, and positive peer relationships can have a positive impact on children’s engagement in learning (Fredricks et al., 2004). Therefore, peer attachment can play a significant mediating role between parent–child relationships and learning adaptability.

Firstly, the study verified the positive effect of the parent–child relationships on peer attachment. In other words, the better the relationship with their parents, the higher the level of attachment to their peers, and the more harmonious their relationship with their peers. Specifically, left-behind children with good parent–child relationships are able to feel the care and love of their parents through free and harmonious parent–child communication, despite their long separation from their parents, so that they can face life with a positive attitude (Acar et al., 2022). Children who grow up in such a harmonious relationship feel emotionally secure and have a positive attitude towards their environment. In addition, this positive attitude to life inspires children to be enthusiastic and confident with their peers, making them more affectionate with their peers and more likely to develop a sense of attachment to them (Nock, 2009). Conversely, left-behind children with poor relationships with their parents are somewhat hindered in their development of relationships with their peers. When parents neglect their children’s education and friendships, they are unable to provide timely and effective guidance on their children’s friendship behavior, which affects the formation of peer relationships (Wen and Lin, 2012). At the same time, a positive family atmosphere and loving behavior among family members are essential for children’s development in all areas, which can lead to positive attitudes toward life and thus to the development of peer interaction skills.

Secondly, the study found that peer attachment was a positive predictor of learning adaptability for left-behind children. According to existing research, learning adaptability plays a crucial role in educational settings, predicting higher classroom participation, as well as higher academic achievement and higher life satisfaction (Burns et al., 2018). As an important relationship for children’s social competence, cognitive and emotional development, peer relationships can reduce the anxiety and isolation that adolescents experience during the particular period when their parents are working outside the home (Liu, 2013). Therefore, peers, as an important part of adolescents’ social relationships, can easily become the object of emotional attachment for left-behind children and alleviate the negative effects of poor parent–child relationships on children (Bogaerts et al., 2006). According to self-determination theory, individuals are able to seek experiences that satisfy their own basic needs through their interactions with the environment. At the same time, the external environment can facilitate the formation of an individual’s motivation to pursue his or her growth and development, and when an individual’s basic needs are met, he or she can effectively combine external experiences with internal motivation and grow (Deci and Ryan, 2000). When left-behind children perceive the care and support of their peers, problems such as the unmet basic psychological needs caused by the lack of family emotions can be resolved. With their basic psychological needs met, children are able to positively change their attitudes towards learning, thus developing their learning abilities and improving their academic performance (Ansong et al., 2017).

In consequence, parents of left-behind children should focus on their parenting behavior and attitudes and pay attention to the family education of left-behind children. On the basis of a harmonious parent–child relationships, children should be guided to establish good peer relationships, which in turn will lead to the development of positive attitudes towards learning and the stimulation and maintenance of motivation in order to provide support and assistance for their children’s academic life.

### 4.2. The moderating role of separation duration

The study found that the findings provided support for the moderating effect of separation duration on parent–child relationships and peer attachment after controlling for gender and grade level, validating hypothesis 3. It was also found that the effect of parent–child relationships on peer attachment decreased as the separation duration increased, according to the moderating effect test. In other words, the effect of the parent–child relationships on peer attachment is greater for children who have been left-behind for a short period of time than for those who have been left-behind for a longer period of time (Hao and Cui, 2007), which is similar to the results of existing studies.

According to existing research, parental neglect of children’s education and friendships can have a negative impact on the formation of harmonious peer relationships (Wen et al., 2019). Therefore, for children who have been left-behind for a short period of time, the short period of emotional absence from the family and the parents’ greater knowledge of their children’s peer interactions makes them more comfortable in guiding their children in their relationships. At the same time, the parent–child relationships remains stronger because of the short period of separation, and the parent–child relationships is more harmonious. In such cases, the family’s problem-solving, communication, emotional response, and behavioral control skills remain strong, which enables children to interact with their peers in a positive and optimistic manner, thus facilitating the development of peer relationships. However, for children who have been left-behind for a long time, the prolonged absence of parental care will have a negative impact on their development (Zhao et al., 2017), predisposing them to a strong sense of isolation (Jia and Tian, 2010) and high social anxiety (Su et al., 2013). Strong feelings of loneliness and high social anxiety tend to create low self-esteem in children, which results in little interaction with others and makes it difficult for children to build good peer attachments. This would reduce the role of parent–child relationships in influencing peer attachment.

## 5. Practical implications

By focusing on the group of left-behind children, the study has enriched the scope of research on children’s learning adaptability, which is of great significance to the change of children’s learning attitudes and the improvement of their learning abilities. Firstly, a harmonious parent–child relationships can improve the learning adaptability of left-behind children. Therefore, parents who work outside the home can make use of information society tools to take various measures to actively communicate with their children and make up for the lack of parent–child communication among left-behind children, so as to establish a good parent–child relationships. Secondly, positive parent–child relationships can facilitate the adaptive development of left-behind children through good peer attachments. Therefore, it is important for parents to strengthen the guidance of children’s interaction behavior and help children to build better relationships with their peers, thus promoting their learning adaptability. Finally, the length of separation duration can affect children’s growth and development. In conclusion, parents can not only reduce the impact of the absence on their children’s development by reducing the amount of time they spend away from them, but can also compensate for the emotional detachment by increasing support for related needs (Moè et al., 2020).

## 6. Limitations and future research

However, there are still several major limitations of the current study. Firstly, the cross-sectional design of the study may have problems such as cohort effects; secondly, the study did not explore the effects of the duration of the child’s initial stay and the frequency of parent–child communication on his or her development; and finally, the study did not explore the moderating effect of separation duration on the relationship between parent–child and academic adjustment and the effect of siblings and others on children’s achievement motivation. In future studies, consideration could be given to including longitudinal studies to understand the effects of early development on children’s behavioral habits. Furthermore, siblings could also be added as a possible moderating influence to provide insight into the role of family composition on children’s achievement motivation. On the other hand, variables such as the frequency of parent–child communication and the time when children start to stay behind can be included in the measurement model, and the effect of the separation duration on the parent–child relationships and learning adaptability can be explored in order to more comprehensively explore the mechanism of the parent–child relationships on the learning adaptability of left-behind children.

## 7. Conclusion

This study investigated the unexplored relationship between parent–child relationships and learning adaptability among Chinese left-behind children by measuring a mediated model with moderation. The model focuses on the mediating role of peer attachment in the relationship between parent–child relationships and learning adaptability and the moderating role of separation duration left-behind in the relationship between parent–child relationships and peer attachment. It is found that (1) parent–child relationships positively predicts children’s learning adaptability; (2) peer attachment, as a mediating variable between parent–child relationships and learning adaptability, positively influences children’s learning adaptability; (3) separation duration plays a moderating role between parent–child relationships and peer attachment, and the positive predictive effect of the parent–child relationships on peer attachment decreases as the separation duration increases. The positive predictive effect of the parent–child relationships on peer attachment gradually decreases as the separation duration increases.

## Data availability statement

The original contributions presented in the study are included in the article/supplementary material, further inquiries can be directed to the corresponding authors.

## Ethics statement

The studies involving human participants were reviewed and approved by the Ethics Committee of Qufu Normal University. Written informed consent to participate in this study was provided by the participants' participants legal guardian/next of kin.

## Author contributions

NC designed the study, helped with data collection, analysed the data. KZ and I-HC designed the study, analysed the data, and wrote the manuscript. GL helped with data analysis and edited the manuscript. All authors contributed to the article and approved the submitted version.

## Funding

This research was supported by the General Project of the National Social Science Foundation of China [grant number 20BSH052].

## Conflict of interest

The authors declare that the research was conducted in the absence of any commercial or financial relationships that could be construed as a potential conflict of interest.

## Publisher’s note

All claims expressed in this article are solely those of the authors and do not necessarily represent those of their affiliated organizations, or those of the publisher, the editors and the reviewers. Any product that may be evaluated in this article, or claim that may be made by its manufacturer, is not guaranteed or endorsed by the publisher.
